# Psychologic interventions in patients with the chronic dermatologic itch in atopic dermatitis and psoriasis: A step forward with family constellations seminars

**DOI:** 10.3389/fmed.2022.965133

**Published:** 2022-08-12

**Authors:** Szergej Capec, Martin Petrek, Gabriella Capec, Roman Yaremkevych, Yuriy Andrashko

**Affiliations:** ^1^Department of Pathological Physiology, Faculty of Medicine and Dentistry, Palacký University, Olomouc, Czechia; ^2^Department of Skin and Venereal Diseases, Faculty of Medicine, Uzhhorod National University, Uzhhorod, Ukraine

**Keywords:** psychological distress, atopic dermatitis, psoriasis, chronic itch, family constellations

## Abstract

Chronic itch is a complex psychophysiological sensation, which can severely affect the quality of life in patients with atopic dermatitis and psoriasis. Itch depends on the irritation of receptors in the skin and the processing of sensory information in the central nervous system. Severe itch leads to activation and later on to disruption of the stress response, resulting in disorders of skin repair, functional and microstructural changes in the areas of the central nervous system that are responsible for the perception of itch. Psychosocial stress can be an essential factor, activating neurohumoral mechanisms which lead to increased itch and scratch, exacerbating skin damage. Patients with chronic itch often have sleep disorders, increased irritability, and depletion of the nervous system. They are characterized by disrupting social relationships, high incidence of anxiety, depressive disorders, and suicidal tendencies. Psychological methods of intervention can effectively influence various mechanisms in the pathogenesis of itch and scratch and improve social functioning in patients with chronic dermatological itch. In this mini-review, we discuss family constellation seminars as an effective method of psychological intervention that can reduce the intensity of itch, and improve sleep and performance in patients with atopic dermatitis and psoriasis. This method is insufficiently described in previous reviews of psychological interventions in atopic dermatitis and psoriasis patients. The positive impact of family constellations seminars in patients with chronic dermatological itch may be related to reducing stress by improving understanding of the family situation, appropriate management of family secrets, and enhancing interactions with the social environment.

## Introduction

Itch is an unpleasant sensation leading to a desire to scratch. Normally, itch and scratch help get rid of parasites or dirt and prevent additional skin damage. In atopic dermatitis (AD) and psoriasis, itch is chronic and can be severe and exhausting. Patients often develop a vicious circle: skin damage (sometimes even minor) leads to stimulation and sensitization of sensory fibers, itch, and scratching, provoking further skin damage, thus significantly reducing the chances of appropriate skin healing and prolonging itch (1).

There are bi-directional relationships between itch and stress:

1. Itch can initiate stress response (2–4).
2. Stress response through endocrine, immune, nervous, and behavioral mechanisms can exacerbate itch (5–7).

Patients with chronic itch differ from healthy individuals: they experience more stress, mental and sleep disorders, more frequent and severe problems in family relationships (8, 9). The current mini-review discusses central psychophysiological mechanisms of itch-scratch-stress interaction and the possible benefits of psychologic intervention in patients with chronic dermatologic itch with family constellation seminars (FCS).

## Association of dermatological itch, stress, and mental disorders

Itch is an important factor in worsening health-related quality of life (2, 10–12). Patients with dermatoses with itch are more stressed than healthy people or patients with dermatologic disorders without itch (11–15). The more intense itch and skin injury, the higher risk of stress and mental disorders (16, 17). Outpatients of dermatology clinics with moderate or severe itch are 10 times more likely to have depression than patients with the mild itch, regardless of the dermatological cause (18).

Atopic dermatitis and psoriasis are the most common dermatological diseases accompanied by itch. Over 80% of patients in dermatology clinics with AD and psoriasis suffer from chronic itch (19, 20). About half of patients in dermatological clinics with AD have a high level of depressive disorders, which is more than 3.5 times higher than in the general population (5, 21, 22). The level of anxiety in adult patients with AD is positively correlated with the intensity of itch (23). In late adolescence, AD with the itch is associated with suicidal ideation (odds ratio, OR > 3.5), mental stress (OR > 2.5), and mental health problems (OR > 2.5) (24). Psoriasis patients are 1.5–3 times more likely to show depressive symptoms and experience a several times higher prevalence of anxiety symptoms, schizophrenia (OR > 2.5), and suicidal ideation than individuals without psoriasis (25, 26).

## Pathophysiological mechanism of itch

### Peripheral mechanisms

The itch sensation depends on the peripheral stimulation of unmyelinated C-type nerve endings (both nociceptors and specialized itch fibers) and the processing of these impulses in the central nervous system. The main substances that stimulate the activation or modulation of these nerve endings are histamine (*via* H1 receptors in acute pruritus and H4 receptors in chronic pruritus), interleukins (IL) IL-1beta, IL-4, IL-6, IL-13, IL-17A, IL-31, IL-33, IL-35, tumor necrosis factor alpha (TNF-α) (27, 28). Most of these molecules are produced by keratinocytes, T-helper cells, mast cells, macrophages, and neutrophils. Activation of sensory nerve endings leads to the release of substance P and calcitonin-gene-related peptide (CGRP), which can enhance the production of the abovementioned cytokines by mast cells and mononuclear cells leading to a vicious circle of increasing itch.

### Central mechanisms of itch and scratch

Information from the sensory nerve endings enters the spinal ganglion and spinal cord and follows the spinothalamic pathway. This information is then processed in the thalamus, somatosensory cortex, cingulate cortex, medial parietal cortex, insular cortex (IC), motor cortex (29), and basal ganglia (30, 31).

Different areas of the brain have different functions in perceiving and processing information about itch. The somatosensory cortex is mainly responsible for the topical perception of itch and its intensity. Activations of the cingulate cortex are likely associated with cognition/evaluation of itch stimuli and/or the urge to scratch. The medial parietal cortex is associated with memory and attention, and at the same time, it may be partially responsible for the subjective sensations of itch and pain. The posterior insular cortex is associated with awareness of affective body feelings (e.g., pain, cold, thirst) and its activity significantly correlates with the intensity of itch stimuli. Activation of the anterior insular cortex correlates with subjective sensation and unpleasantness of itch. Usually, this part of the brain is considered responsible for awareness of emotions and subjective feelings (29).

### Peculiarities of itch perception in patients with chronic dermatologic itch

Chronic itch patients’ minds are often occupied by negative thoughts due to their itch, including their past unpleasant itch episodes (17). Many of the abovementioned brain areas are activated more intensely in patients with AD than in healthy controls, both with the physical induction of itch on the skin and when watching someone scratch (32). In patients with AD, there is greater activation of the basal ganglia (which are among other functions responsible for motivation and craving) when exposed to histamine on the affected areas of the skin, resulting in excessive itch. In patients with psoriasis, brain structures related to the perception and response to itch demonstrate both functional and microstructural changes (31).

Activity in the right medial prefrontal cortex, posterior cingulate cortex/precuneus, and angular gyrus correlates with the severity of chronic itch (33). These structures are essential in autobiographical memory retrieval, envisioning the future, conceiving the perspectives of others, participate in using past experiences to plan for the future, navigate social interactions (34), and it is important to research if psychological interventions in patients with chronic itch can influence the activity of these structures.

## Basic mechanisms of the stress response

**FIGURE 1:**
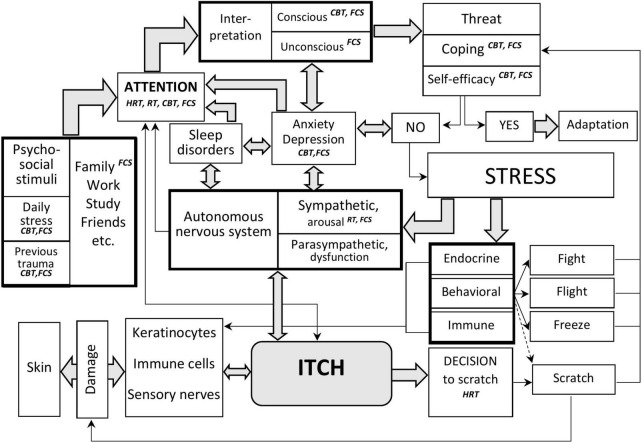
Interaction of stress and itch, key points of psychologic interventions in patients with itch. The perception of itch and the reaction to it depend on the interaction of sensory impulses from the skin, attention to these stimuli, and the state of the autonomic nervous system. Attention is modified under the influence of other external stimuli, including psychosocial, sleep, state of mental functions, etc. After the attention filter, sensory information is processed, and the general adaptation syndrome (stress reaction) is triggered in the case of a threat to survival. The stress reaction modifies endocrine, behavioral, and immune reactions. It can lead to changes in the function of keratinocytes, immune cells, and nerve structures, worsening the skin condition and increasing itching. With the help of psychological methods, it is possible to influence attention, interpretation of stimuli, methods of behavioral reactions, and improve the psychosocial adaptation of patients with chronic pruritus. The psychological techniques discussed in the article and the key points of their influence are indicated in italics. *HRT*, habit reversal training; *RT*, relaxation training; *CBT*, cognitive behavioral therapy; *FCS*, family constellations seminars.

In modern society, most stress reactions are related to the social environment: family, partners, colleagues, and friends. If a person does not have efficient strategies to adapt, general adaptation syndrome (stress) mechanisms are triggered (Figure 1). These mechanisms include activation of the nervous system (autonomous nervous system, cortical and subcortical structures), the endocrine system (primarily the hypothalamus-pituitary-adrenal axis), behavioral mechanisms (fight-flight-freeze reactions), and the immune system (35). The response by scratching can be one of the equivalents of the desire to get rid of an unpleasant stimulus, not only from a physical one but also in symbolic form, from a mental trigger.

## Itch and stress interactions in dermatologic patients

### Itch as a stressor

Intensive itch is an independent factor that triggers a stress response (4). Patients with chronic itch experience neurophysiologic irritation, which can be an additional factor in the emergence of conflicts, stresses, mental disorders, and finally in depletion of endocrine mechanisms of the stress response (36). Peak levels of daily stressors are associated with an increase in psoriasis severity a month later and a lower cortisol level (37).

### Skin changes under acute and chronic stress

Acute and chronic mental stress affects the skin in many ways (7). Keratinocytes have receptors for catecholamines, histamine, acetylcholine, neurotrophic factors, glucocorticoids, and neuropeptides (e.g., substance P and nerve growth factor) (6). Keratinocytes and fibroblasts locally produce hormones that are traditionally attributed to the hypothalamic-pituitary system, namely, corticotropin-releasing hormone, proopiomelanocortin, and molecules that occur during its degradation: ACTH, opioid hormones, alpha-melanocyte-stimulating hormone (7, 38). In moderate acute stress, the barrier function of the skin and its regeneration may improve (4). However, under severe acute psycho-emotional stress inflammatory processes in the skin of patients with psoriasis are activated. TNF-α, sympathetic nervous system, and neuropeptide system are essential players in this activation. Mental stress increases the production of IL-6 by keratinocytes, changes the composition of the secretion of sebaceous glands, and as a consequence, leads to damage of the barrier function of the skin (38). The effects of chronic stress include a long-term increase in endogenous glucocorticoids associated with impaired skin regeneration and permeability, leading to exacerbation of itch. However, a significant proportion of patients with AD have an insufficient systemic hormonal response to acute stress, which may be a sign of the depletion of adaptive mechanisms (36).

### Role of itch and stress in the development of sleep disorders

Both itch and psychosocial conflicts can disrupt patients’ sleep (39). Multidirectional relationships exist between pruritus intensity and psychological distress, psychological distress and sleep disturbances, and pruritus intensity and sleep disturbances (3, 40). Sleep disorders can result in additional irritability, impaired social functioning, and a higher risk of mental disorders in patients with chronic pruritus. Even one night of sleep deprivation leads to increased levels of glucocorticoids, and along with acute stress, impairs the barrier function of the skin, increases skin dryness, itch, and worsens the course of AD (4).

### Social interactions are important causes of stress in patients with chronic dermatologic itch

Leading sources of stress in patients with AD and psoriasis include social interactions and itch itself (2, 8, 41). Patients with AD and psoriasis are often characterized by a high level of childhood traumatic events, physical neglect in childhood (42), stressful life events, higher anxiety and depression scores (12, 43), long-lasting family distress (9, 44), family secrets, emotional abuse, alcohol and drug abuse (8), insecure attachment styles (45, 46) inability to perceive safety environment (14) and helplessness (2). Many dermatologic patients with chronic itch feel a pleasurable sensation after scratching even in the absence of itch (29), so they may use scratching as a way to soothe themselves to decrease stress. Both adults and adolescents with atopic dermatitis point to the importance of psycho-emotional factors in triggering the subjective feeling of itch, followed by scratching (47).

### Social loyalty, agreeableness, and alexithymia may be associated with impaired social functioning in patients with chronic itch

Atopic dermatitis and psoriasis can be perceived as a disorder of communication between an individual and the environment (48). Many psoriatic and AD patients cannot reach personal goals in social situations. They refuse to acknowledge the presence of the high family strain even when there are objective signs and causes of family stress (14, 20). Among possible causes of this phenomenon can be high family loyalty (20). Psoriasis patients describe themselves as more cooperative and agreeable than healthy controls. In psoriasis patients, public self-consciousness is significantly positively associated with induced itch, and agreeableness is significantly negatively associated with induced scratching (49). High levels of alexithymia may be another essential mechanism explaining misinterpretation of social situations and emotional distress by patients with AD. More than 56% of patients with AD have alexithymia, compared with 21% of healthy individuals in the control group (21). Alexithymia, in turn, can develop as a reaction of “fading” to reduce maladaptation and exhaustion with a large number of stresses. Patients with pruritus also tend to develop dissociative states (in which the psyche is detached from bodily sensations), which are typical for psychological trauma (2). In patients with chronic itch, dissociative states may be one of the ways to reduce subjective discomfort.

## Psychological interventions in patients with itch

Medications, biological therapy, and psychological interventions are essential to control itch (27, 50). In our own work, we focus on the psychological mechanisms of influence on itch, and describe the possible mechanisms of influence of the method of FCS (51) in detail, and compare this method with other forms of psychological interventions.

Several types of psychological interventions were effective in the reduction of itch and scratch in patients with psoriasis and AD (52–55). According to an older meta-analysis (56), autogenous training (AT), cognitive behavioral therapy (CBT), dermatological education and CBT, and stress management program significantly decreased itch in these patients. AT, CBT, dermatological education and CBT, and habit reversal training (HRT) effectively decreased scratching intensity and eczema severity, though HRT did not decrease the feeling of itch. After a one-month course of relaxation therapy (RT), patients with AD demonstrated reduced itch and improved sleep quality compared with the control group (23). Objectively, the skin condition in patients of the RT group on the background of basic therapy improved in the same way as in the control group, but the level of biomarkers did not change (23).

Atopic dermatitis and habit reversal training do not address family problems as an essential source of stress. They are based on targeting attention, arousal, and scratching behavior, which is a relatively late stage in the itch-scratch process (Figure 1). At the same time, proper understanding of the social situation, successful coping strategies (57), acting with awareness (58), feeling of self-efficacy, and appropriate attachment orientation (46) may be critical in the reduction of stress by CBT and FCS interventions.

## Family constellation seminars as a candidate method for patients with chronic itch

Family constellation seminars are becoming increasingly popular in solving various problems related to relationships and health (59). The number of English literature publications explaining the basics of this method and the peculiarities of use in different groups of patients is limited (59, 60). Recent randomized control clinical trials (61, 62) have shown that FCS effectively help people manage family-related psychological issues especially connected with implicit interactions and family secrets. Participants of FCS reported significant improvement in psychological functioning, psychological distress, decreased motivational incongruence, better experience in their personal social systems, and overall goal attainment after FCS and in 4- and 12- follow-up periods after FCS (62, 63).

Family constellation seminars method was not discussed in several reviews about psychological interventions in the patients with itch (52–56), but we reported its effectiveness in decreasing itch, scratch, and improving skin condition (51). After a series of FCS, along with reduced itch severity and duration, patients with AD and psoriasis improved attentiveness, working capacity, productivity, quality of sleep and daily activities, and felt less emotional depletion due to itch (51).

**FIGURE 2:**
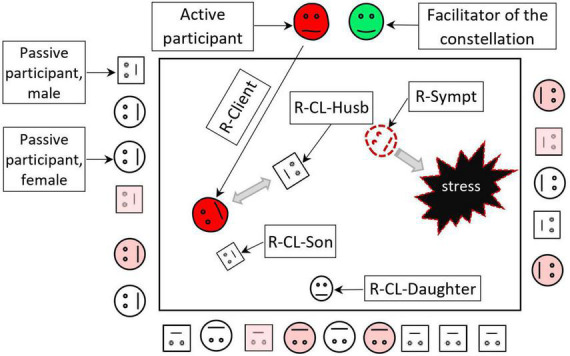
Scheme of the family constellation session. Family constellations are usually held in groups of 20–25 participants. A client who wants to solve his problem (active participant) chooses representatives from other participants for the roles of himself, his symptom, and his family members. After the roles are assigned, the client places the representatives according to the roles in the room as real family members. Subconsciously, representatives experience various emotions, bodily sensations, and thoughts similar to those of real members of the client’s family system with reasonable accuracy. After that, the facilitator helps interpret the participants’ psychological interactions in the constellation and makes interventions to improve the interactions between the client, his symptom, and his family members. As a result of the constellation, the client receives a new model of more harmonious interaction in the family. Gray arrows show interactions. Representative of a symptom often looks at the stressful event. Seminar participants (square figures–males, circles–females) observe what is happening in the client’s family system. Some of them (marked in pink) can respond emotionally and take models for solving their family problems. R-Client, representative of a client; R-CL, daughter–representative of the client’s daughter; R-CL-Husb, representative of the client’s husband; R-CL-Son, representative of the client’s son; R-Sympt, representative of the client’s symptom.

Family constellation seminars are usually held in group sessions with about 20–25 participants and are led by a trained facilitator, usually a psychotherapist or clinical psychologist (63). The facilitator often uses the genogram to diagnose implicit family stresses and relationships, and that is of particular importance in patients with chronic itch (8, 20). With the help of the facilitator, one of the participants (the client, the so-called “active participant”) describes essential facts about his family and his problem. After that client asks other participants to act as representatives (or so-called “stand-ins”) for him/herself and his/her family members to depict the actual family interactions. Active participant places representatives in the room according to his/her own image of the stressful situation (Figure 2). Under the supervision of the facilitator, representatives subconsciously interact as if they were real people from the client’s life. The difference between the FCS method and other group interaction methods is that after the placement in the specific places, representatives act according to their subconscious impulses depicting the behavior and emotions of family members with reliable accuracy. The facilitator makes phenomenological interpretations of the active participant’s and representatives’ cognitive, emotional and bodily reactions, and implements psychological interventions, helping the active client understand additional information about the family situation, developing more balanced interactions. After interventions, representatives change their places, and finally, the optimal “solution constellation” should provide a new, more comfortable pattern of the family relationships for the client. In the “solution constellation,” the client is able to communicate and behave more efficiently in his/her personal social system (59).

Family constellation seminars postulates that a symptom (e.g., itch) may often play an adaptive role in the functioning of the individual and his/her family as a social unit, so the active participant often assigns a representative to depict his symptom or disease in the constellation (64). The representative in the role of a symptom often helps to reveal important information about the specific psychological trauma or pattern of stressful relationships in the family. In “solution constellation,” the representative of the symptom usually feels that he/she is no longer needed to balance a client’s system. By acquiring the new information and the new models of behavior, not only do active participant feels more resourceful, but all the participants of FCS use the new experience to improve real interactions with their family members (64).

The family constellations approach can also be used in individual (private, face-to-face) form and videoconferencing. Private setting differs from the group context, particularly with regard to the representatives: in groups, the participants themselves are used, while in an individual session, the constellation is done with specifically designed figurines, objects, or pictures (60).

## Possible benefits of the family constellation seminars method for patients with chronic itch

1. Family constellation seminars addresses both conscious and unconscious family communication issues, helping reveal family secrets (especially connected with severe psychologic trauma) and making sense of implicit interactions between family members.
2. Usage of representatives and observation of their interactions from the third person point of view (in dissociated mode) may help the patient decrease stress from traumatic events revealed during the constellation. Considering the tendency of dermatologic patients to avoid speaking about family problems (20), this feature of FCS can be of essential importance.
3. Participation both as active participants and as representatives in the process of a constellation of the other participants of the seminar helps clients learn adaptive and safe models of behavior in their stressful situations, improving self-efficacy and coping skills (61, 62), especially important for patients with helplessness and worrying (2). Considering that patients with itch have difficulties with consciously admitting family problems (20), FCS might decrease stress in patients by offering a new solution to the problem without placing extra responsibility on the clients.
4. Family constellation seminars is traditionally held in groups of approximately 25 people, helping to establish a safe microenvironment (61) and possibly improve the attachment style of dermatologic patients.
5. Family constellation seminars is economically efficient due to the group format and the possibility of the weekend and online settings (61, 62).
6. From 66 to 92% of FCS participants reported increased happiness, courage, optimism, higher coping abilities, and improved interpersonal relationships due to the intervention (59). It is essential for patients with chronic itch, who are at an increased risk of depression and anxiety.
7. Family constellation seminars has a long-lasting effect, which may be of special importance in patients with chronic dermatoses (51, 62).

In conclusion, this review provides evidence for the effective use of FCS for stress coping in a general population sample and in patients with a variety of mental health disorders. Based on pathophysiological and social aspects of chronic itch and our own experience, we suggest implementing this method in the dermatological clinical setting. However, prior to its wider implementation, data from further research on the applications of FCS and other forms of family-centered psychologic interventions in patients with chronic itch are required.

## Author contributions

SC, MP, and GC performed the literature review, designed the figures, and wrote the manuscript. RY and YA added intellectual content and critically revised the manuscript. All authors approved the final manuscript for publication.
